# Acetate and propionate effects in response to LPS in a porcine intestinal co-culture model

**DOI:** 10.1186/s40813-023-00316-y

**Published:** 2023-05-23

**Authors:** Melania Andrani, Paolo Borghetti, Francesca Ravanetti, Valeria Cavalli, Luca Ferrari, Elena De Angelis, Paolo Martelli, Roberta Saleri

**Affiliations:** Department of Veterinary Science, University of Parma, Strada del Taglio 10, 43126 Parma, Italy

**Keywords:** SCFA, IPEC-J2, PBMC, Tight junctions, Co-culture system, Intestinal epithelial barrier function

## Abstract

**Background:**

The interest in acetate and propionate as short chain fatty acids (SCFA) derives from research on alternative strategies to the utilization of antibiotics in pig farms. SCFA have a protective role on the intestinal epithelial barrier and improve intestinal immunity by regulating the inflammatory and immune response. This regulation is associated with an increase in intestinal barrier integrity, mediated by the enhancement of tight junction protein (TJp) functions, which prevent the passage of pathogens through the paracellular space. The purpose of this study was to evaluate the effect of in vitro supplementation with SCFA (5 mM acetate and 1 mM propionate) on viability, nitric oxide (NO) release (oxidative stress), NF-κB gene expression, and gene and protein expression of major TJp (occludin [OCLN], zonula occludens-1 [ZO-1], and claudin-4 [CLDN4]) in a porcine intestinal epithelial cell (IPEC-J2) and peripheral blood mononuclear cell (PBMC) co-culture model upon LPS stimulation, through which an acute inflammatory state was simulated.

**Results:**

Firstly, the inflammatory stimulus induced by LPS evaluated in the IPEC-J2 monoculture was characterized by a reduction of viability, gene expression of TJp and OCLN protein synthesis, and an increase of NO release. The response evaluated in the co-culture showed that acetate positively stimulated the viability of both untreated and LPS-stimulated IPEC-J2 and reduced the release of NO in LPS-stimulated cells. Acetate also promoted an increase of gene expression of CLDN4, ZO-1, and OCLN, and protein synthesis of CLDN4, OCLN and ZO-1 in untreated and LPS-stimulated cells. Propionate induced a reduction of NO release in both untreated and LPS-stimulated IPEC-J2. In untreated cells, propionate induced an increase of TJp gene expression and of CLDN4 and OCLN protein synthesis. Contrarily, propionate in LPS-stimulated cells induced an increase of CLDN4 and OCLN gene expression and protein synthesis. PBMC were influenced by acetate and propionate supplementation, in that NF-κB expression was strongly downregulated in LPS-stimulated cells.

**Conclusions:**

The present study demonstrates the protective effect of acetate and propionate upon acute inflammation by regulating epithelial tight junction expression and protein synthesis in a co-culture model, which simulates the in vivo interaction between epithelial intestinal cells and local immune cells.

**Supplementary Information:**

The online version contains supplementary material available at 10.1186/s40813-023-00316-y.

## Background

Short-chain fatty acids (SCFA) such as acetate, propionate, and butyrate are produced during anaerobic fermentation of undigested carbohydrates and fiber polysaccharides by the intestinal microbiota in the proximal large intestine of pigs [1, 2]. Propionate derives from succinate through the metabolism of carbohydrates (glycolysis), while butyrate and acetate derive from acetyl-CoA through glycolysis [3]. Total SCFA concentration in the large intestine varies depending on the diet, with a molar ratio of 3:1:1 for acetate, propionate, and butyrate [2].

Fatty acids have been considered an alternative to antibiotics or zinc oxide [4] as they stimulate immunity by triggering local proliferation and the adaptive immune response [5], particularly against *Salmonella enteritidis* and enteropathogenic *Escherichia coli* [6, 7]. SCFA are pivotal as a fuel for intestinal epithelial cells and as antibacterial agents, inducing cell lysis and inhibition of enzyme activity [8], as well as stimulating gastrointestinal motility [9]. SCFA improve the intestinal barrier function, reduce apoptosis of epithelial cells, increase intestinal gene expression and protein synthesis in the mucosa, and sustain intestinal development and growth in weaned piglets [10]. The gastrointestinal tract is strongly exposed to a variety of pathogens, and changes in the gut microbial composition can cause diarrhoea, particularly during the weaning and fattening periods when piglets are exposed to new oral antigens [11]. A balanced communication between intestinal epithelial cells (IEC) and immune cells ensures the maintenance of a healthy and immunologically protective physical barrier through the secretion of inflammatory signals, such as cytokines and chemokines, and the expression of Toll-like receptors which interact with conserved pathogen-associated molecular patterns (PAMP) [12]. IEC regulate the permeability of adjacent cells through tight junction proteins (TJp), including occludin (OCLN), claudins, and zonula occludens-1 (ZO-1) [13]. In turn, ZO-1 interacts with the cytoplasmic tails of occludin and claudins.

The alteration of the barrier caused by pathogens or their toxins (e.g., bacterial lipopolysaccharide, LPS) and the consequent increase of paracellular permeability can induce activation of the mucosal immune system, resulting in prolonged inflammation and tissue damage [14]. It is fundamental for the intestinal barrier to maintain its integrity through the presence of specific proteins, such as claudins, occludins, and zonula occludens.

To evaluate the interplay between IEC and immune cells and to mimic the physiological barrier function at the intestinal level, we previously characterized a co-culture model [15]. The model was based on the co-culture of a IEC swine cell line (IPEC-J2) [16] and peripheral mononuclear cells (PBMC) [15].

By using this cell model, the objective of this study was to evaluate how acetate and propionate influence intestinal barrier integrity, in the presence of an LPS-induced inflammatory stimulus in intestinal porcine epithelial cells (IPEC-J2) through the influence on cell viability, the release of nitric oxide (NO) and the regulation of TJp.

## Results

### Cell viability

**Fig. 1:**
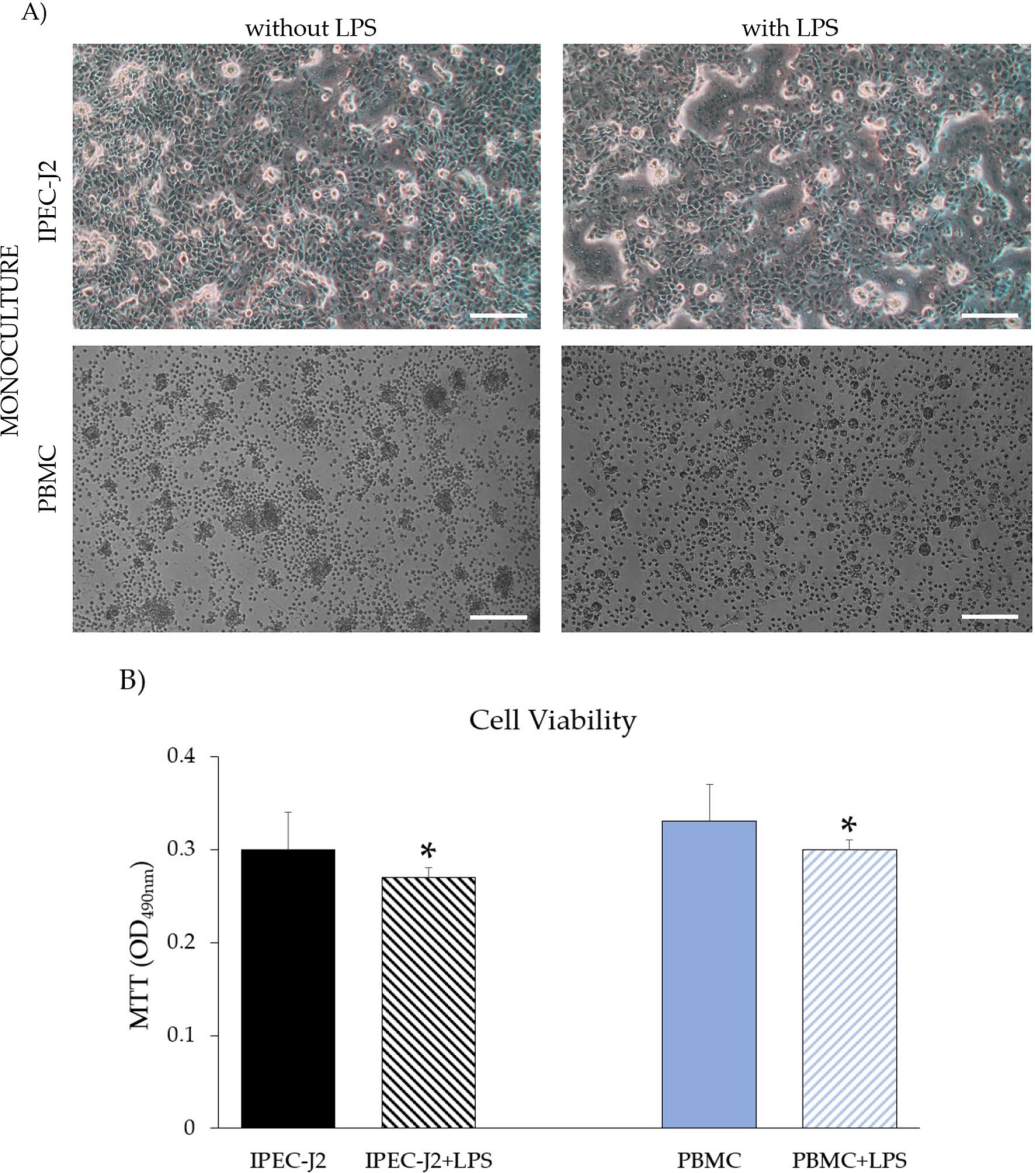
**A** Light microscopy of monocultures: IPEC-J2 and PBMC with or without LPS; 4 × original magnification, scale bar = 50 μm. **B** Viability of IPEC-J2 and PBMC monocultures upon LPS stimulation. Each value represents the mean ± SD of 8 replicates of 6 independent experiments. Asterisks indicate significant differences (*p* < 0.05) upon comparison with the respective control (IPEC-J2 or PBMC) without LPS

As can be observed in Fig. 1A, IPEC-J2 in the monoculture control group showed a confluent monolayer while control PBMC (without LPS) previously stimulated with PHA showed evident morphofunctional rosettes formation. Compared to the control group, upon stimulation with LPS, IPEC-J2 and PBMC showed a decrease in cell number. The morphology of IPEC-J2 was less elongated, and the rosettes in PBMC were reduced in size (Fig. 1A). Figure 1B shows that a significant decrease of viability occurred in IPEC-J2 and PBMC monocultures in the presence of LPS (*p* < 0.05).

**Fig. 2:**
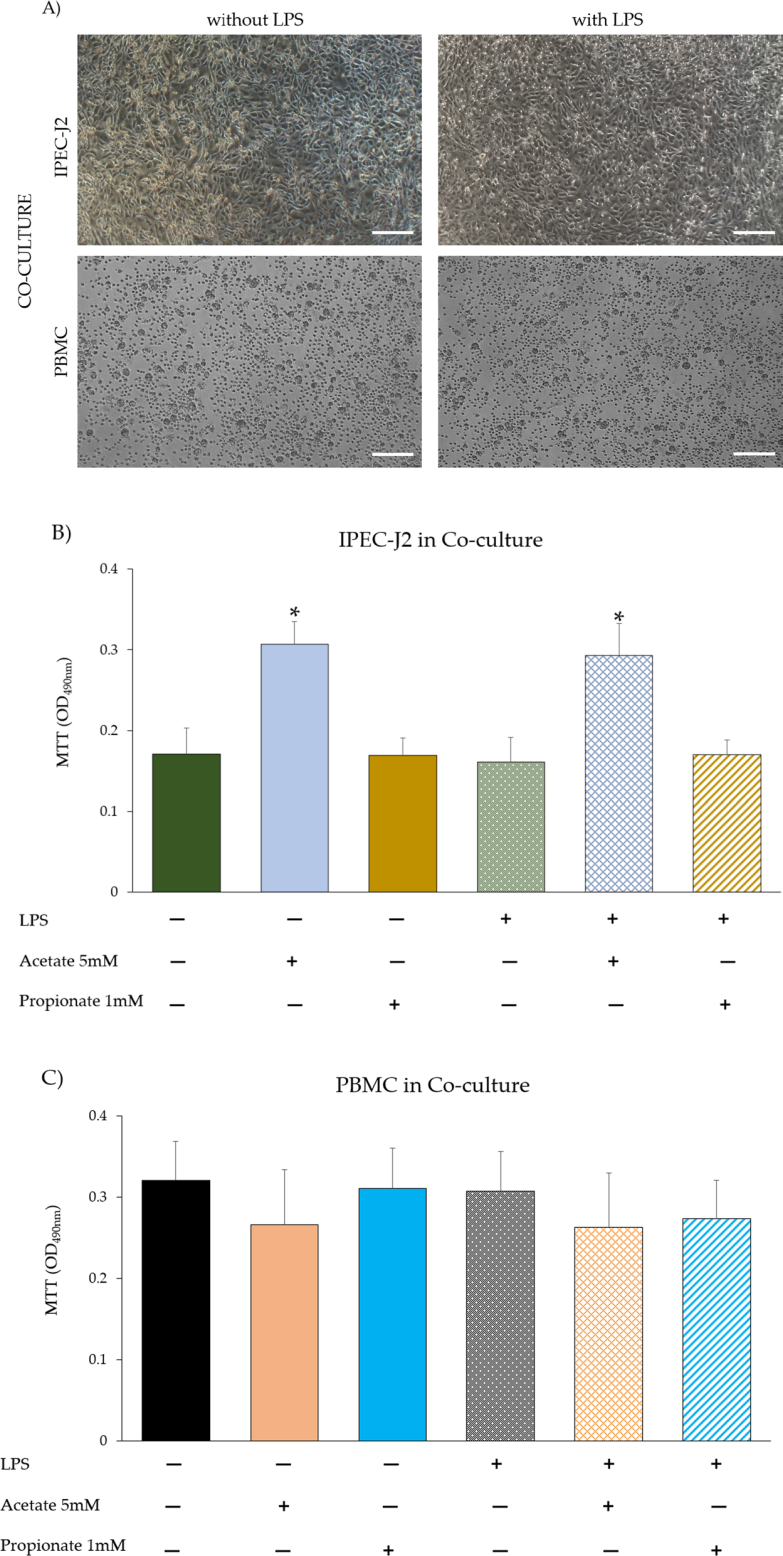
**A** Light microscopy of co-cultures: IPEC-J2 (transwell) and PBMC (well) with or without LPS; 4 × original magnification, scale bar = 50 μm. **B** Cell viability of IPEC-J2 and **C** PBMC in co-culture with or without LPS and medium supplementation with acetate (5 mM) or propionate (1 mM) at 24 h of incubation was determined using an MTT assay. Each value represents the mean ± SD of 8 wells of 6 independent experiments. Significant differences (*p* < 0.05) among groups are indicated by asterisks

In the co-culture, IPEC-J2 viability was not significantly influenced by LPS (Fig. 2A, B). PBMC viability with or without acetate or propionate supplementation, and upon LPS did not show significant changes (Fig. 2A, C). Upon acetate treatment, cell viability increased in IPEC-J2 (*p* < 0.05) compared to untreated control cells (Fig. 2B). However, no significant differences were detected in all other IPEC-J2 and PBMC groups (with/without LPS and/or with propionate) as compared to the respective control (Fig. 2B, C).

### Nitric oxide (NO)

**Fig. 3:**
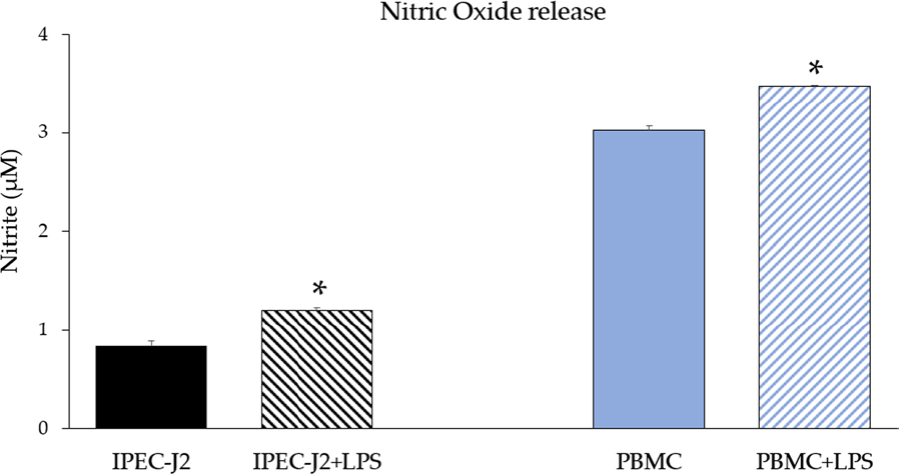
Nitric oxide release (quantified as nitrite) in supernatants of IPEC-J2 and PBMC monocultures upon LPS stimulation. Each value represents the mean ± SD of 8 replicates of 6 independent experiments. Asterisks indicate significant differences (*p* < 0.05) upon comparison with the respective control (IPEC-J2 or PBMC) without LPS

**Fig. 4:**
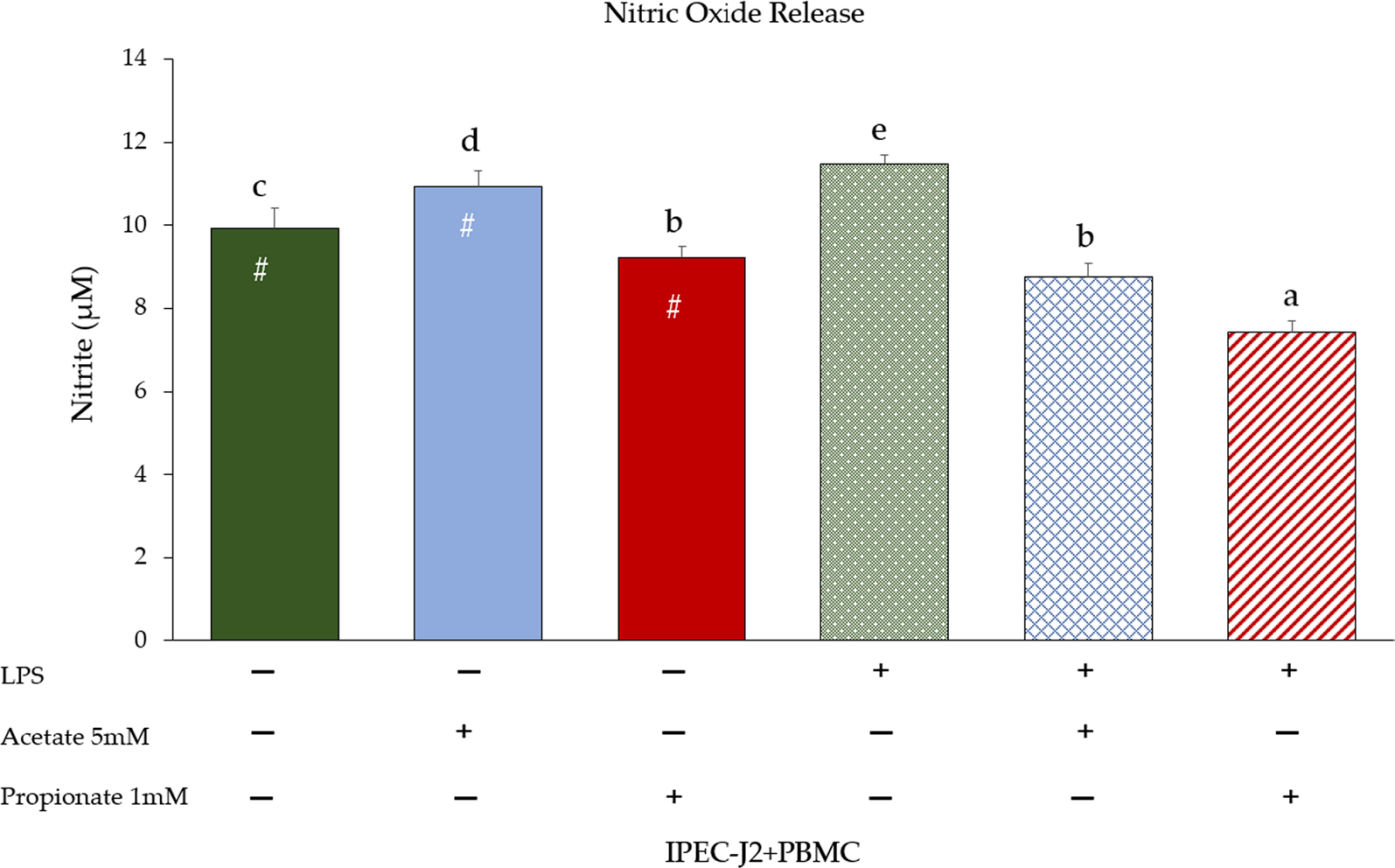
Effect of co-culture conditions (acetate or propionate treatment) with or without LPS on NO release after 24 h of incubation. Each value represents the mean ± SD of 8 wells of 6 independent experiments. Significant differences (*p* < 0.05) between each LPS-untreated group and the corresponding LPS-treated group are indicated with a hashtag (#). Different letters indicate significant differences among groups (*p* < 0.05)

Data regarding NO release (quantified as nitrite) in the supernatants of monocultures are presented in Fig. 3. Both in IPEC-J2 and PBMC, NO release significantly increased upon LPS stimulation (*p* < 0.05). A significant increase of NO release in untreated co-culture compared to monocultures was observed (*p* < 0.05) (Fig. 4). LPS in the co-culture stimulated an increase of NO production (*p* < 0.05). Acetate treatment induced NO release, whereas propionate treatment showed a slight decrease of NO (*p* < 0.05). In contrast, both SCFA treatments (especially propionate) induced a reduction of NO release upon LPS stimulation (*p* < 0.05).

### Gene expression and protein levels in monocultures

**Fig. 5:**
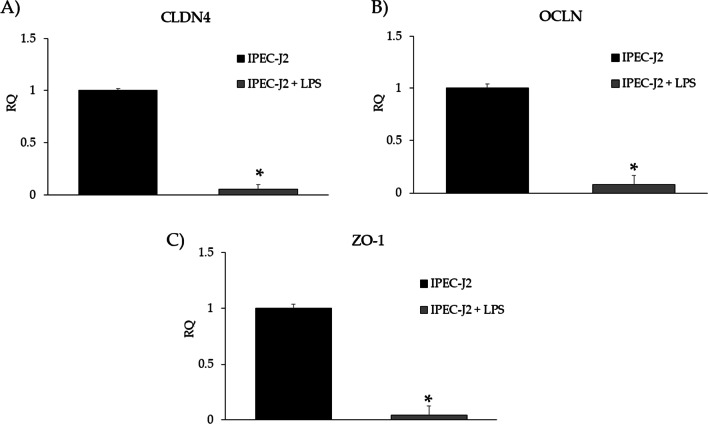
**A** CLDN4, **B** OCLN, and **C** ZO-1 gene expression in IPEC-J2 monoculture upon LPS stimulation. Each value represents the mean ± SD of 8 replicates of 6 independent experiments. Asterisks indicate significant differences between groups (*p* < 0.05). Data were analyzed using the 2^−ΔΔCt^ method, in which the expression levels of the gene, normalized to the expression of the reference gene HPRT1, were expressed as relative quantities (RQ). The analysis was performed by defining IPEC-J2 as reference group

**Fig. 6:**
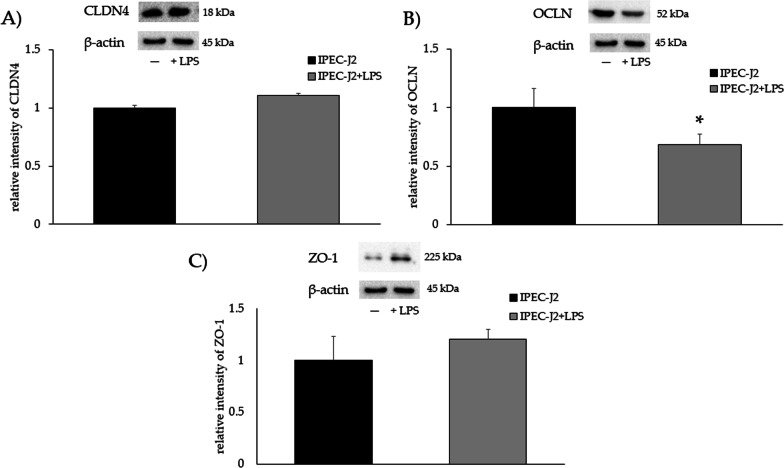
**A** CLDN4, **B** OCLN, and **C** ZO-1 protein levels in IPEC-J2 monoculture upon LPS stimulation. Each value represents the mean ± SD of 8 replicates of 6 independent experiments. Asterisks indicate significant differences between groups (*p* < 0.05). Data were normalized to the reference protein β-actin as relative intensity. The analysis was performed by defining IPEC-J2 as reference group. The samples derived from the same experiment and the gels/blots were processed in parallel. The original blots are presented in Additional file 1: Figure S6A, B, C

Data reported in Fig. 5 show gene expression of TJp in the IPEC-J2 monoculture. CLDN4 (Fig. 5A), OCLN (Fig. 5B) and ZO-1 (Fig. 5C) expression levels were significantly reduced upon LPS stimulation compared to controls (IPEC-J2) (*p* < 0.05). The protein levels of CLDN4, OCLN and ZO-1 determined by western blotting are shown in Fig. 6A–C. While CLDN4 and ZO-1 protein levels did not show significant changes upon LPS stimulation, OCLN protein levels significantly decreased (*p* < 0.05).

### Tight junction protein (TJp) gene expression and protein levels in IPEC-J2 co-cultured with PBMC and LPS

**Fig. 7:**
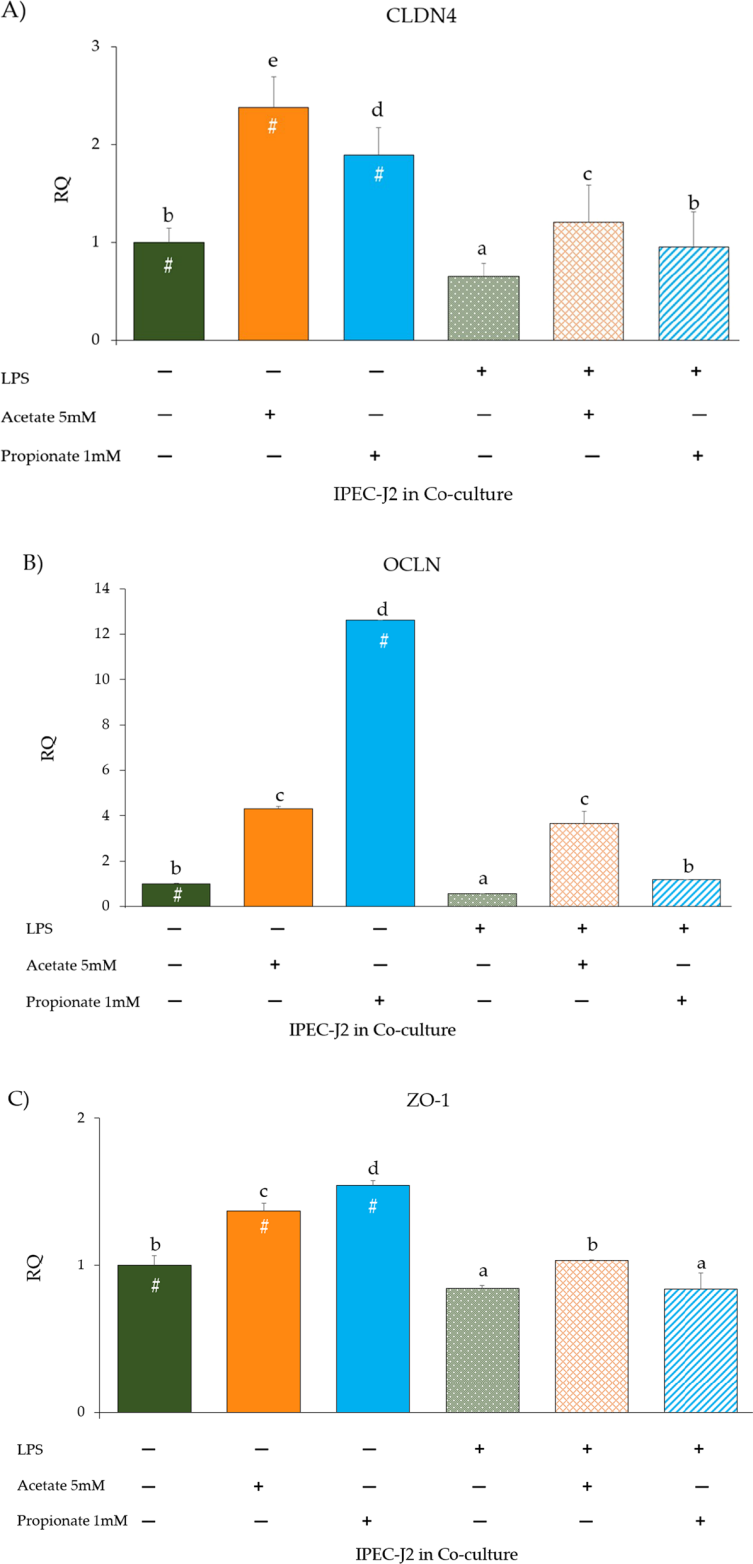
**A** CLDN4, **B** OCLN, and **C** ZO-1 gene expression in IPEC-J2 co-cultured with PBMC with or without LPS and/or acetate or propionate treatment. Each value represents the mean ± SD of 8 replicates of 6 independent experiments. Significant differences (*p* < 0.05) between each LPS-untreated group and the corresponding LPS-treated group are indicated with a hashtag (#). Different letters indicate significant differences among groups (*p* < 0.05). Data were analyzed using the 2^−ΔΔCt^ method, in which the expression levels of the gene, normalized to the expression of the reference gene HPRT1, were expressed as relative quantities (RQ). The analysis was performed by defining untreated IPEC-J2 + PBMC as reference group (first histogram of each graph)

**Fig. 8:**
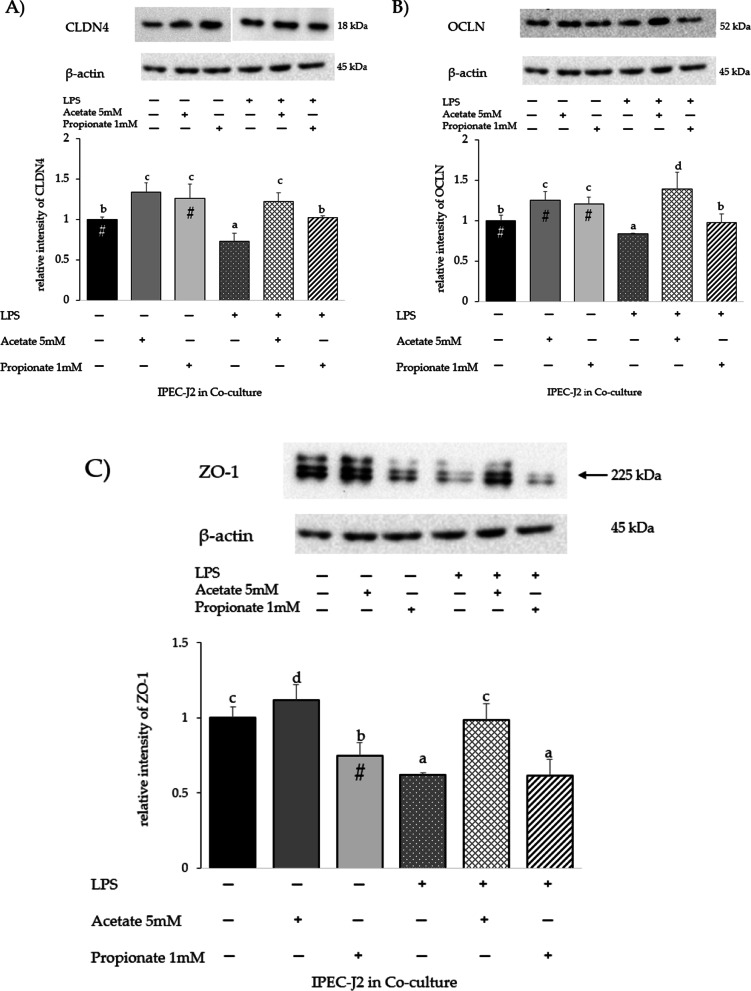
**A** CLDN4, **B** OCLN, and **C** ZO-1 proteins in IPEC-J2 co-culture with PBMC with/without LPS and/or acetate or propionate treatment. Each value represents the mean ± SD of 8 replicates of 6 independent experiments. Letters indicate significant differences among groups (*p* < 0.05). Significant differences (*p* < 0.05) between each LPS-untreated group and the corresponding LPS-treated group are indicated with a hashtag (#). Data were normalized to the synthesis of the reference protein β-actin as relative intensity. The analysis was performed by defining untreated IPEC-J2 + PBMC as reference group (first histogram of each graph). The samples derived from the same experiment and the gels/blots were processed in parallel. The original blots are presented in Additional file 1: Figures S8 A, B, C

TJp gene expression was investigated in IPEC-J2 co-cultured with PBMC (Fig. 7A–C). CLDN4, OCLN, and ZO-1 expression significantly increased upon acetate and propionate treatment compared to the untreated co-culture control (*p* < 0.05). Significant increases of CLDN4, OCLN, and ZO-1 expression were observed upon acetate treatment in LPS-stimulated cells compared to LPS stimulation alone (*p* < 0.05). In contrast, in the presence of propionate and LPS, ZO-1 expression was significantly reduced compared to untreated control (*p* < 0.05) (Fig. 7C). In addition, as shown in Fig. 8, the protein levels of CLDN4, OCLN, and ZO-1 were significantly reduced in the presence of LPS. Acetate supplementation in untreated IPEC-J2 and in LPS-stimulated cells induced an increase of CLDN4, OCLN and ZO-1 levels (*p* < 0.05) (Fig. 8A–C). A comparable increase of CLDN4 and OCLN was observed upon propionate supplementation in untreated cells and LPS-stimulated cells compared to controls (*p* < 0.05) (Fig. 8A, B).

### Nuclear factor-κB (NF-κB) expression in PBMC co-cultured with IPEC-J2 and LPS

**Fig. 9:**
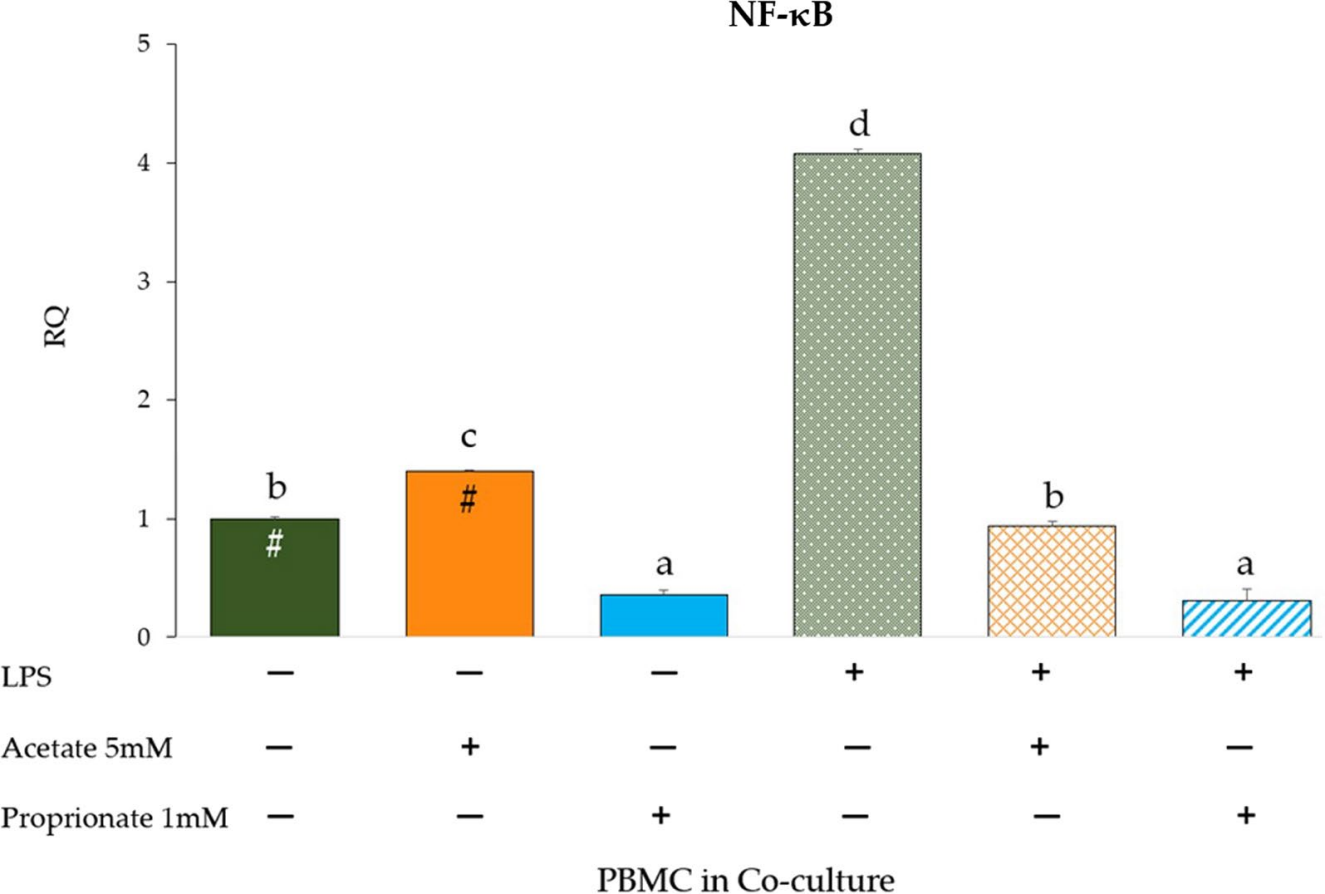
NF-κB gene expression in PBMC co-cultured with IPEC-J2 with or without LPS and/or acetate or propionate treatment. Each value represents the mean ± SD of 8 replicates of 6 independent experiments. Significant differences (*p* < 0.05) between each LPS-untreated group and the corresponding LPS-treated group are indicated with a hashtag (#). Different letters indicate significant differences among groups (*p* < 0.05). Data were analyzed using the 2^−ΔΔCt^ method, in which the expression levels of the gene, normalized to the expression of the reference gene HPRT1, were expressed as relative quantities (RQ). The analysis was performed by defining untreated IPEC-J2 + PBMC as reference group (first histogram of the graph)

Data reported in Fig. 9 show gene expression of NF-κB in PBMC under co-culture conditions. NF-κB gene expression significantly increased upon acetate treatment compared to PBMC in the untreated co-culture control (*p* < 0.05) while opposite effect was observed with propionate. A remarkable increase of NF-κB expression was observed upon LPS stimulation compared to control (*p* < 0.05). Notably, both acetate and propionate induced a strong reduction of NF-κB expression in LPS-stimulated co-culture (*p* < 0.05).

## Discussion

The intestinal epithelium allows nutrient absorption and acts as a barrier, preventing antigens and pathogens from entering the mucosal tissues. The epithelial junctional adhesion complex plays an important role in the intestinal barrier. Among the different mechanisms by which pathogens can interact with intestinal cells, they can contribute to barrier impairment by downregulating epithelial tight junctions [17].

SCFA may play a key role in intestinal disease prevention by modulating multiple effects on immune cells during intestinal inflammation and by providing energy to the IEC, which in turn respond by redistributing TJ and activating mucosal immune cells able to secrete cytokines and chemokines. These reactions lead to a reduction of intestinal disorders in the early stages of life of pigs under stressful conditions (e.g., weaning, social interactions, feed changes) [18].

In the present study, an acute inflammatory condition was induced by LPS stimulation, which had a significant effect on IPEC-J2 and PBMC monocultures. In co-cultures not supplemented with SCFA, the negative LPS effect on IPEC-J2 and PBMC viability was not observed, thus suggesting a positive inter-communication between the two cell types.

While in monocultures LPS mainly induced a significant down-regulation of TJp gene expression, in co-cultures LPS stimulation induced an overall decrease of protein synthesis of CLDN4, OCLN and ZO-1. The reduction of TJp gene expression in this model may have been mitigated by a positive effect due to PBMC, which however may not have supported protein synthesis. Therefore, this possibly led to increased epithelial permeability and in turn intestinal barrier dysfunction, supported by the increase of NO. Nitric oxide is the end-product of an important molecular signalling pathway for the induction of gut barrier failure; its regulation is fundamental in various physiological processes as a secondary messenger and inflammatory modulator [19]. Although the release of NO is correlated with oxidative stress, representing a potential negative condition, acetate supplementation alone in co-culture induced an increase of NO production and a concomitant increase of IPEC-J2 viability. Indeed, some studies support that a low amount of NO synthesized by constitutive nitric oxide synthase has a protective role in the maintenance of intestinal barrier integrity [20]. In addition, the NO increase could be explained by the effect of acetate on an *ex novo* synthesis of arginine, which is both an essential amino acid that favours cell viability/proliferation and a precursor of NO [21]. Indeed, under LPS-induced inflammatory conditions, acetate favoured a protective effect, by reducing NO release and inducing IPEC-J2 to proliferate; therefore, NO release could be influenced by the PBMC response. Also PBMC were influenced by acetate supplementation, as this SCFA can be uptaken by immune cells, where it upregulates glycolysis and subsequently improves immune functions [22]. Compared to acetate treatment alone, upon acetate supplementation in LPS-stimulated cells, a strong reduction of NF-κB expression in PBMC was observed, which is essential for the induction of iNOS gene expression [23].

Conversely, in our model, intestinal epithelial cells and PBMC viability/proliferation were not affected by propionate treatment alone or upon LPS stimulation. In a previous study [24], cell viability/proliferation of IPEC-J2 monoculture was induced by 1 mM propionate; in our co-culture model, the concentration of this SCFA may not be sufficient to induce a proliferative effect on both cell types in the co-culture. Propionate could have a protective effect, even upon LPS stimulation, as we observed a reduction of NO release associated with a strong downregulation of NF-κB expression.

The effect of SCFA on cell viability and NO production may be related to alterations of TJp [25]. Upon inflammation, CLDN4 reduction is associated with a decrease in cytoplasmic ZO-1 and transmembrane OCLN, causing a damage to the epithelial barrier characterized by functional alterations, loss of polarity due to ion mislocalization, dysregulation of nutrient absorption/secretion [26], changes in cell proliferation and differentiation, and gene expression [27].

In our co-culture model, acetate significantly induced the expression and synthesis of TJp, particularly of OCLN and CLDN4, thus affecting the epithelial barrier function, and this modulation was related to increased cell viability. Differently, even if propionate supplementation induced the major expression of OCLN in untreated IPEC-J2, it had a lesser effect on LPS-stimulated cells. OCLN is suggested to have less influence than CLDN4 on barrier integrity [28]; in our conditions, this may be associated to the absence of changes in cell proliferation.

The supplementation with acetate or propionate showed a comparable effect upon LPS stimulation; we could consider their protective role on the epithelial intestinal barrier due to the stimulation of TJp synthesis. We can speculate that this protective effect is attributable to the inhibition of histone deacetylase (HDAC), through which SCFA increase the acetylation of histone and non-histone proteins from chromatin, including NF-κB, thereby modulating gene expression of this transcription factor, as observed in this study, and other genes [29].

In summary, the functionality of the IPEC-J2/PBMC co-culture model was confirmed upon LPS stimulation in terms of cellular responsiveness to the inflammatory stimulus both as gene expression and protein synthesis. Our results suggest that acetate and propionate reduce LPS-induced NO production in intestinal epithelial and immune cells and strengthens cell-to-cell contacts by increasing TJp on the membrane, which could limit the negative effect of an acute inflammatory condition.

In addition, our model based on the communication between IPEC-J2 and PBMC highlighted the modulation of the physical defense due to the tight junctions, which membrane expression is reduced in the presence of LPS. The model can mimic the activation of the mucosal immune response which in vivo is characterized by the recruitment of immune cells via chemokines, cytokines, and growth factors [12].

## Conclusions

Few studies have investigated the effects of acetate and propionate on inflammation. The intestinal co-culture model used in the present study suggests that acetate and propionate have protective effects on the physical barrier against acute inflammation. In addition, this model supports the feasibility of investigating the impact of and interaction between SCFA and the complex intestinal environment in vitro.

## Materials and methods

### Cell cultures

IPEC-J2 cells (cell line derived from the jejunum of an unsuckled neonatal pig) were cultured in flasks in Dulbecco’s Modified Eagle Medium/Ham’s F-12 (DMEM/Ham’s F-12) (Merck; Darmstadt, Germany) + 5% fetal bovine serum (FBS) (ThermoFisher; Waltham, MA, USA), supplemented with penicillin (100 U/mL), streptomycin (100 μg/mL), amphotericin B (0.5 µg/mL) and glutamine (2 mM) (Merck; Darmstadt, Germany) in a humidified environment at 37 °C, 5% CO_2_. The number of IPEC-J2 was determined using a haemocytometer, and cell viability (never less than 95%) was evaluated using Trypan blue (0.1%) (Merck; Darmstadt, Germany) exclusion. After 24 h, at approximately 80–90% confluence, IPEC-J2 were trypsinized and seeded on the top surface of collagenized cell culture inserts (0.3 cm^2^ polyethylene terephthalate membrane with 0.4 μm pore size) (Costar, Corning Inc., Corning, NY, USA) at a density of 1 × 10^5^ cells/cell culture insert. Cells were used between passage 28 and 30. PBMC were obtained from the blood of 10-month-old pigs from a slaughterhouse certified by the Italian Ministry of Health according to Regulation (EC) 853/2004 (Sassi S.P.A., Parma, Italy; approval nr. CE-IT-190-M). The cells were isolated by Histopaque-1077® (Merck) density gradient and viability was checked by Trypan blue (Merck) (never less than 90%). Cells were washed twice with DMEM/Ham’s F-12, seeded at 5.5 × 10^6^ cells/well in 24-well plates and stimulated for 24 h with 5 μg/mL phytohemagglutinin (PHA).

### LPS stimulation of IPEC-J2 and/or PBMC in co-culture

After 24 h of culture, IPEC-J2 in transwells were centrifuged, moved to the plate where PBMC were seeded, and the supernatants from all wells were changed with DMEM/Ham’s F-12 + 5% FBS. To trigger an inflammatory response in the whole co-culture system, LPS (1 μg/mL) was added and the cells were incubated at 37 °C and 5% CO_2_ for 24 h. Simultaneously, cells were incubated with SCFA supplementation (5 mM acetate or 1 mM propionate) or in the absence of any supplementation for 24 h, as reported below. The experimental groups for IPEC-J2 or PBMC in monoculture were IPEC-J2 or PBMC in DMEM/Ham’s F-12 medium (IPEC-J2 or PBMC), or with LPS (IPEC-J2 + LPS or PBMC + LPS).

The experimental conditions for IPEC-J2/PBMC co-cultures in DMEM/Ham’s F-12 were as follows:

1. untreated or treated with LPS;
2. treated with acetate 5 mM or propionate 1 mM;
3. treated with LPS and acetate 5 mM, or treated with LPS and propionate 1 mM.

The ranges of SCFA concentrations were chosen based on data reported in our previous study [24].

### IPEC-J2 viability assay

At the end of both incubations for 24 h, the viability of IPEC-J2 and PBMC was evaluated under monoculture and co-culture experimental conditions (presence/absence of LPS and/or 5 mM acetate or 1 mM propionate). Cell viability was determined by Trypan blue exclusion and using a 3-(4,5-dimethylthiazol-2-yl)-2,5-diphenyltetrazolium bromide (MTT) colorimetric assay (Merck). Briefly, IPEC-J2 and PBMC were seeded into 24-well plates at a density of 1 × 10^5^ cells/well and 5.5 × 10^6^ cells/well, respectively, for 24 h in 1 mL of complete medium, or in medium supplemented with LPS and/or SCFA. MTT assays were performed by adding 20 μL (5 mg/mL) of an MTT solution to IPEC-J2 and PBMC, followed by incubation for 4 h. Afterwards, the medium was removed and IPEC-J2 and PBMC were lysed with 150 μL dimethyl sulfoxide (DMSO) (Merck) to solubilize the purple formazan crystals. After resuspension, the lysate was transferred to a 96-well plate and detected at 490 nm using a VICTOR® Nivo™ Multimode Microplate Reader (PerkinElmer, Waltham, MA, USA).

### Nitric oxide (NO) assay

NO production was assessed by measuring the amount of nitrite (NO_2_^–^), a stable metabolic product of NO, in cell culture supernatants, by the Griess reaction after 24 h of incubation for all cell culture conditions using a VICTOR® Nivo™ Multimode Microplate Reader (Perkin Elmer), as previously reported [24].

### RNA extraction and reverse transcription (RT)

Total RNA was isolated from an 8-well pool using a EuroGold Trifast™ kit solution (Euro-clone, Milan, Italy) according to the manufacturer’s instructions and reverse-transcribed to generate complementary DNA (cDNA) using oligo-dT primers (Bioneer; Daejeon, Korea); purity (260/280 nm ratio) and concentration (at 260 nm) were assessed using a BioSpectrometer® (Eppendorf AG, Hamburg, Germany). RNA samples were treated with DNAse (Merck), and 1 µg/20 µL was reverse-transcribed using HiScript® III RT SuperMix (Vazyme Biotech Co.; Nanjing, China). RT was performed using a StepOne™ thermocycler (Applied Biosystems, StepOne™ software v.2.3) according to the manufacturer’s instructions under the following thermal conditions: 2 min at 45 °C, 15 min at 37 °C, followed by 5 s at 85 °C. The cDNA samples were stored at − 20 °C.

### Real-time quantitative PCR (qPCR)

The cDNA samples were used as templates for qPCR, which was performed using a StepOne™ thermocycler (Applied Biosystems, StepOne™ software v.2.3). cDNA (20 ng/20 µL) was amplified in duplicate using Fast PowerUp™ SYBR™ Green Master Mix (Applied Biosystems; Foster City, CA, USA) and specific primer sets (Eurofins Genomics, Ebersberg, Germany) at 400 nM for ZO-1 and at 300 nM for the other genes. Details of each primer set are presented in Table 1. Samples were maintained at 95 °C for 20 s and then subjected to 40 cycles consisting of a denaturation step at 95 °C for 3 s, followed by an annealing/extension step at 60 °C for 30 s. Data were analyzed using the 2^−ΔΔCt^ method [30], in which the expression levels of each gene were normalized to the reference gene hypoxanthine phosphoribosyltransferase-1 (HPRT-1) [25, 30]. The reference HPRT-1 gene was selected among other tested reference genes (i.e., β-2MG, GAPDH, and 18S rRNA) as the endogenous control according to minimal intra-/inter-assay variation. cDNA amounts expressed as relative quantities (RQ) were calculated related to the expression level in IPEC-J2 or PBMC in DMEM/Ham’s F-12 medium at 24 h for monocultures and in IPEC-J2 + PBMC in DMEM/Ham’s F-12 medium at 24 h for co-cultures. A melting curve analysis for the specific amplification control was performed (60–95 °C) at the end of the amplification cycles. No-RT controls and no-template controls (NTC) were included, and the latter was assumed to be negative and reliable if Ct was ≥ 35.

**Table 1:** Target genes and primer sequences used for SYBR Green qPCR

Target gene	GenBank Accession nr	Primer sequence	Efficiency (%)	Slope	r^2^	Amplicon length (bp)
CLDN4 [31]	AB235916	F 5′-TATCATCCTGGCCGTGCTA-3′ R 5′-CATCATCCACGCAGTTGGT-3′	100.2	− 3.29	0.99	71
OCLN [32]	FN400888	F 5′-GGAGTGATTCGGATTCTGTCTATGCT-3′ R 5′-CGCCTGGGCTGTTGGGTTGA-3′	103.8	− 3.03	0.99	423
ZO1 [32]	XM_021098896	F 5′-GGCGCACGGCGAAGGTAATT-3′ R 5′-CTATCAAACTCAGGAGGCGGCACT-3′	103.7	− 3.21	0.98	405
HPRT-1 [31]	DQ845175	F 5′-ACACTGGCAAAACAATGCAA-3′ R 5′-TGCAACCTTGACCATCTTTG-3′	102.0	− 3.25	0.99	71
GAPDH [15]	NM_001206359	F 5′-GGTGAAGGTCGGAGTGAACG-3′ R 5′-GCCAGAGTTAAAAGCAGCCCT-3′	102.0	− 3.27	0.99	70
β-2MG [33]	DQ17123	F 5′-AAACGGAAAGCCAAATTACC-3′ R 5′-ATCCACAGCGTTAGGAGTGA-3′	106.3	− 3.19	0.98	178
18S rRNA [34]	AY265350.1	F 5′-CCCACGGAATCGAGAAAGAG-3′ R 5′-TTGACGGAAGGGCACCA-3′	100.9	− 3.30	0.99	125
NF-κB [31]	DQ834921	F 5′-GAAGGACCTCTAGAAGGCAAAA-3′ R 5′-GCTTTGGTTTATGCGGTGTT-3′	99.6	− 3.21	0.99	125

HPRT-1 was used as endogenous reference gene

*HPRT-1* hypoxanthine phosphoribosyltransferase 1, *CLDN4* claudin 4, *OCLN* occludin, *ZO-1* zonula occludens, *β-2MG* β-2-microglobulin, *GAPDH* glyceraldehyde-3-phosphate dehydrogenase, *rRNA* ribosomal RNA, *NF-κB* nuclear factor-κB, *bp* base pairs

### Western blotting analysis

IPEC-J2 proteins were extracted by lysis of cells in cold buffer (10 mM Tris–HCl, pH 8; 10 mM NaCl; 3 mM MgCl_2_; 0.1% SDS; 0.1% Triton X-100; 0.5 mM EDTA) containing protease inhibitor mix (10 µg/mL APMSF; 0.5 µg/mL Leupeptin; 0.7 µg/mL Pepstatin), and protein concentration was measured using the Pierce BCA Protein assay Kit (ThermoFisher Scientific, Waltham, MA, USA). Equal concentrations of proteins (20 µg) were heated at 100 °C for 5 min and then separated by 10% SDS-PAGE in TRIS buffer. A PageRuler pre-stained protein ladder (ThermoFisher Scientific, Waltham, MA, USA) was used as the molecular weight standard. After electrophoresis, the proteins were transferred to a PVDF membrane (Hybond–P; Amersham, Buckinghamshire, UK), and the membrane was blocked in PBS + 0.05% Tween (PBST) with 5% skim milk for 1 h, at room temperature, and incubated with the same buffer containing a specific primary antibody: claudin-4 1:500 (#32-9400; Invitrogen, Carlsbad, CA, USA); occludin 1:2,000 (#33-1500; Invitrogen, Carlsbad, CA, USA); zonula occludens-1 1:2000 (#PA5-28858; Invitrogen); β-actin 1:2000 (#4970S; Cell Signalling; Danvers, Massachusetts, USA) overnight, at 4 °C. After repeated washing with PBST buffer, HRP-conjugated secondary antibody [HRP-conjugated goat anti-rabbit IgG 1:50,000 (#31460 ThermoFisher); HRP-conjugated goat anti-mouse IgG 1:50000 (#31430 ThermoFisher)] was applied for 1 h at RT. Visualization was performed using a chemiluminescent enzyme substrate (SuperSignal West Pico Plus #34580; ThermoFisher Scientific, Waltham, MA, USA). The signal intensities of specific bands were detected using the ChemiDoc™ MP Imaging System (Bio-Rad) and quantified using Image J software (National Institutes of Health, Bethesda, MA, USA). β-actin was used as a control for equal loading, and the results are expressed as the optical density of the target protein/β-actin.

### Statistical analysis

Statistical analysis was carried out using SPSS (IBM® SPSS® Statistics v.28, NY, USA). The normal distribution of the data was assessed by the Shapiro–Wilk test and the comparison between treated and untreated samples was performed by one-way ANOVA using a model with groups and interaction between groups as fixed factors. The least significant difference (LSD) post-hoc test was used to compare means when significant differences (*p* < 0.05) were found. Experimental data are presented as mean ± standard deviation. Differences among groups were considered significant at *p* < 0.05.

## Supplementary Information

**Additional file 1**. Supplementary figures.

## Data Availability

All data are available from the corresponding author upon reasonable request.
