# Supplementary material for: Acetate and propionate effects in response to LPS in a porcine intestinal co-culture model
Source: Porcine Health Manag. 2023 May 23;9:23. doi: 10.1186/s40813-023-00316-y (PMC10207778; doi:10.1186/s40813-023-00316-y)
Supplement: Supplementary file 1 — Additional file 1. Supplementary figures. [file 40813_2023_316_MOESM1_ESM.docx]

**Acetate and propionate effects in response to LPS in a porcine intestinal co-culture model**

Melania Andrani^1*^, Paolo Borghetti^1^, Francesca Ravanetti^1^, Valeria Cavalli^1^, Luca Ferrari^1^, Elena De Angelis^1^, Paolo Martelli^1†^, Roberta Saleri^1†^

^1^ Department of Veterinary Science, University of Parma, Strada del Taglio 10, 43126 Parma, Italy

* Correspondence: melania.andrani@unipr.it

**^†^** These authors contributed equally to this work and share last authorship.

**Supplementary Information**

**Figure S6** A, B, C) β-actin protein levels in IPEC-J2 monoculture upon LPS stimulation. The bands indicated with the asterisks (*) are reported in the main text.


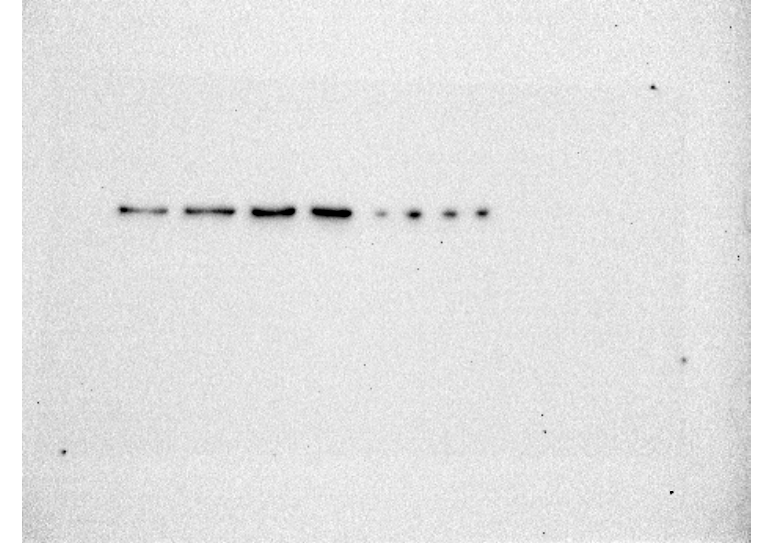


*

*

**Figure S6** A) CLDN4 protein levels in IPEC-J2 monoculture upon LPS stimulation. The bands indicated with the asterisks (*) are reported in the main text.


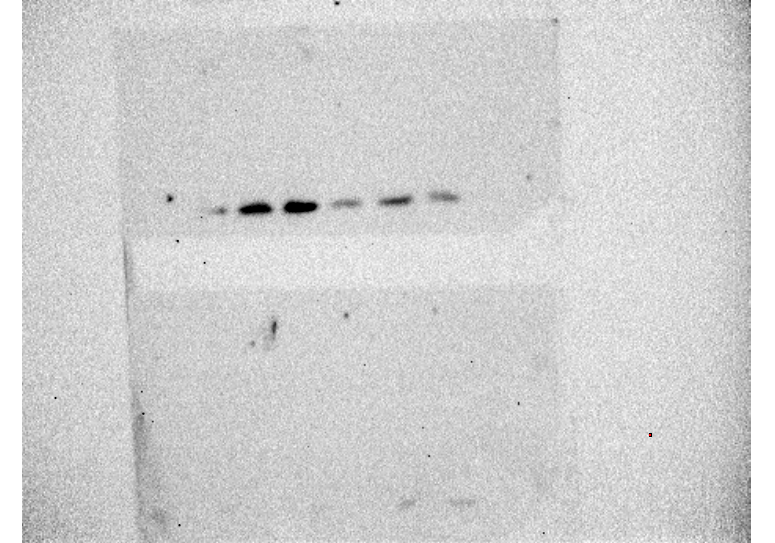


*

*

**Figure S6** B) OCLN protein levels in IPEC-J2 monoculture upon LPS stimulation. The bands indicated with the asterisks (*) are reported in the main text.


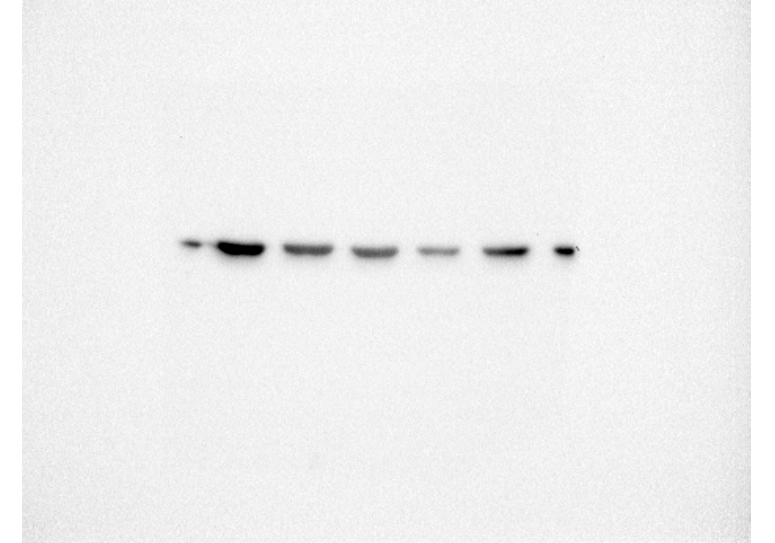


*

*

**Figure S8** A,B,C) β-actin proteins in IPEC-J2 co-culture with PBMC with/without LPS and/or acetate or propionate treatment.


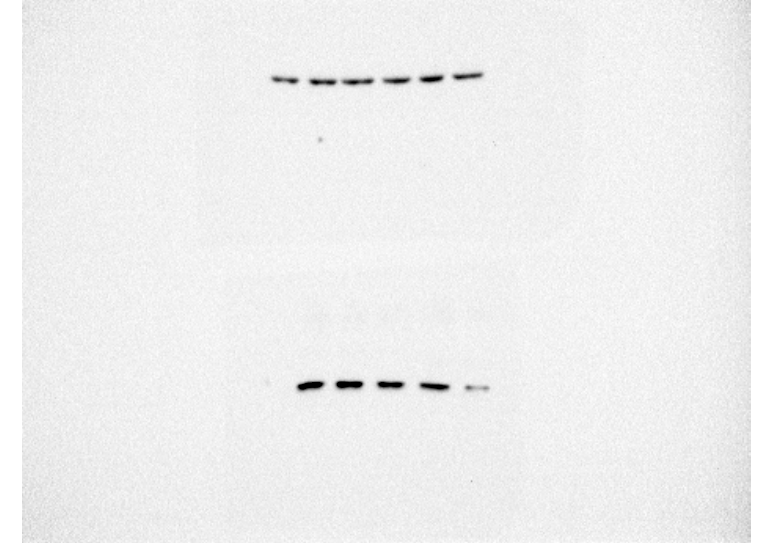


**Figure S8** A) CLDN4 proteins in IPEC-J2 co-culture with PBMC with/without LPS and/or acetate or propionate treatment. The bands indicated with the asterisks (*) are reported in the main text (first three bands).


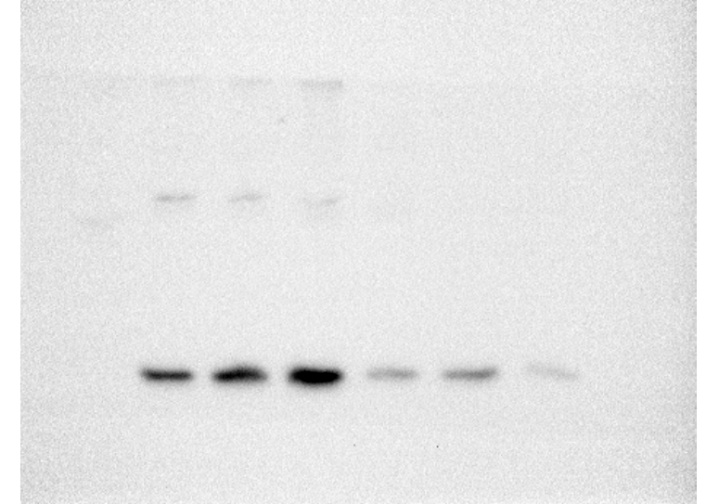


*

*

*

**Figure S8** A) CLDN4 proteins in IPEC-J2 co-culture with PBMC with/without LPS and/or acetate or propionate treatment. The bands indicated with the asterisks (*) are reported in the main text (last three bands).


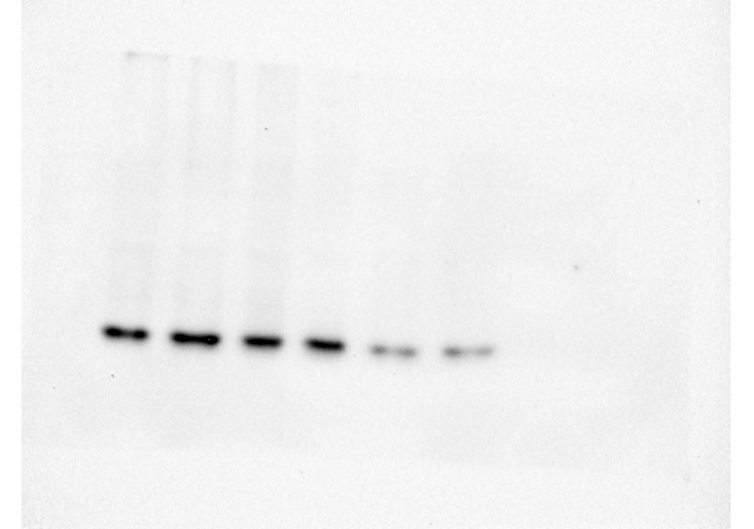


*

*

*

**Figure S8** B) OCLN proteins in IPEC-J2 co-culture with PBMC with/without LPS and/or acetate or propionate treatment.


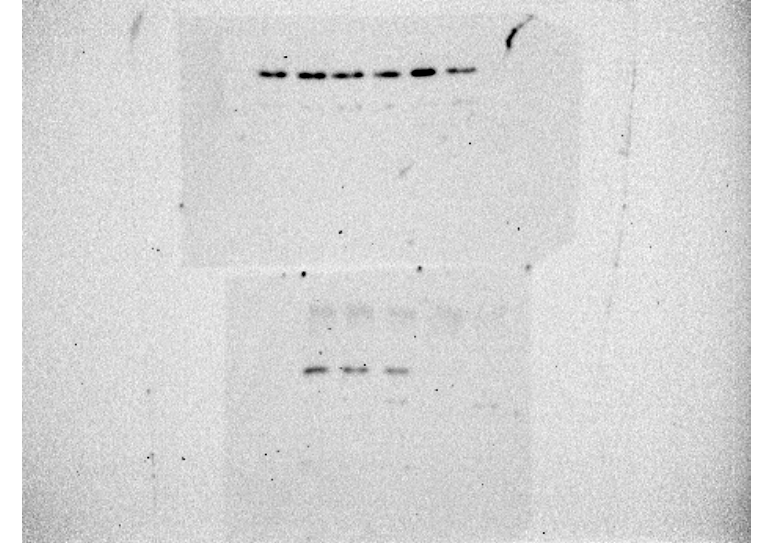


**Figure S6 (monoculture) and Figure 8 (co-culture)** ZO-1 protein levels in IPEC-J2 monoculture and co-culture with PBMC with/without LPS and/or acetate or propionate treatment.


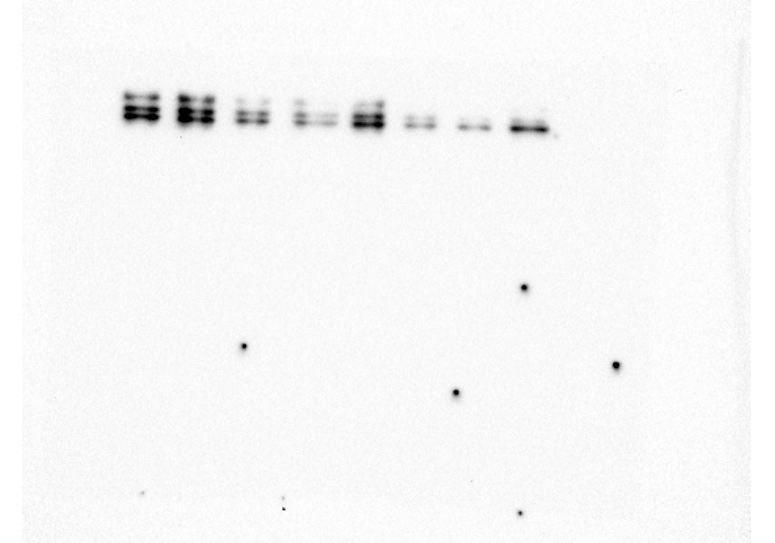


Monoculture

Co-culture
